# Editorial: Psychological distress in healthy, vulnerable, and diseased groups: Neurobiological and psychosocial bases, detection methods, and creative management strategies

**DOI:** 10.3389/fpubh.2023.1185503

**Published:** 2023-04-27

**Authors:** Amira Mohammed Ali, Maha Atout, Rasmieh Al-Amer

**Affiliations:** ^1^Department of Psychiatric Nursing and Mental Health, Faculty of Nursing, Alexandria University, Alexandria, Egypt; ^2^School of Nursing, Philadelphia University, Amman, Jordan; ^3^Faculty of Nursing, Isra University, Amman, Jordan; ^4^School of Nursing and Midwifery, Western Sydney University, Penrith, NSW, Australia

**Keywords:** mental distress, dementia family caregivers, emotional processing, COVID-19 vaccine, loneliness, Depression Anxiety Stress Scale-8, older adults, college students

Psychological distress is described as the non-specific mental symptoms of depression, anxiety, personality traits, and multiple psychological (e.g., burnout), somatic, and behavioral problems. It results from complex dynamics (social, psychological, neurochemical, etc.) associated with overwhelming and sustained stress and painful experiences. The COVID-19-related psychological impact has been widely addressed in this article collection. Nurses are directly involved in COVID-19 care, and two studies have denoted that their physical and mental health may considerably suffer. Alzahrani et al. reported a 63.8 and 68.8% prevalence of depression and anxiety, respectively, among emergency nurses in Saudi Arabia during the COVID-19 pandemic. Significant risk factors associated with anxiety and depression were low physical activity and working in urban areas. Using a purposive sampling approach, Al-Amer et al. interviewed 10 Jordanian nurses from a hospital designated for COVID-19 patients to explore the experience of Arab nurses of caring for COVID-19 patients. The themes generated from the qualitative data uncovered an impact of COVID-19 on nurses' health, unfamiliar work and social environments, and a need to conform to professional standards. The study highlighted specific risks to the physical and mental wellbeing of Arab nurses caring for COVID-19 patients.

Because of pandemic distress, especially among those caring for irreversible and chronic conditions, Ali et al. used data from an online survey of dementia family caregivers to examine the psychometrics of the Depression Anxiety Stress Scale 8-items (DASS-8) relative to two longer distress measures. The DASS-8 demonstrated adequate validity (construct, measurement invariance, convergent, criterion, and discriminant) relative to the DASS-12 and DASS-21. Known-group validity tests revealed greater distress among female caregivers, the adult children of care recipients, those caring for patients with high dependency in their activities of daily living, and those who received help with care (e.g., from health professionals). The DASS-8 revealed higher distress and strongly correlated with loneliness, suggesting its usability for identifying caregivers with greater proneness to psychopathology.

Because of the failure of lockdowns and traditional protective measures against COVID-19, vaccines have been promoted by the WHO to limit infection transmission, leading to many reported concerns regarding their safety and efficacy in different countries around the world. Based on a nationwide survey in Bangladesh, Alam et al. investigated the psychological effects and associated factors among individuals who received or did not receive COVID-19 vaccines. Consistent with similar studies, vaccinated individuals had significantly lower prevalence rates of psychological distress (36.4 vs. 51.5%), depression (21.1 vs. 37.9%), anxiety (25.1 vs. 44.9%), stress (19.4 vs. 30.4%), PTSD (29.4 vs. 38.3%), insomnia (18.7 vs. 39.4%), and fear symptoms (16.1 vs. 27.5%) than those who were not vaccinated, especially those who were employed or living in Dhaka. Among vaccinated people, living in nuclear families and losing family members or friends because of COVID-19 were associated with greater levels of distress, depression, anxiety, fear, and post-traumatic stress symptomatology.

Harboring large numbers of students and teachers, the school environment has been extensively affected by COVID-19-related closures and the strict emphasis on the use of protective measures, which represented a cause of stress for teachers, students, and families. Rǎducu and Stǎnculescu examined the burnout profiles of 330 Romanian kindergarten and primary school teachers and their association with various stressors, including workload, student misbehavior, lack of recognition, and poor colleague relationships. The study uncovered four burnout profiles, with high workload, student misbehavior, and lack of recognition being the main stressors. Better career opportunities, time management, and classroom management could help prevent teacher burnout, especially during the COVID-19 pandemic, which has increased burnout symptoms.

The mental health of university students has also been addressed in many studies. Chen F. et al. studied the psychological mechanisms of English emotion word processing under the semantics–prosody Stroop effect paradigm in quiet and noisy listening environments among Chinese college students with trait depression (TD). Compared with low-TD students, high-TD students displayed a marked lack of sensitivity toward emotions. The Stroop effect influenced emotion word processing automatically, regardless of trait severity or listening conditions. While the speech-shaped noise backdrop had no effect, the participants were more affected by the Stroop effect when doing prosody tasks and recognizing emotions than when performing semantic activities and identifying the valence of English words. The results suggest an emotional processing disadvantage in individuals with high TD and the congruence-induced facilitation effect in the general Stroop effect. In another study, Zhang et al. examined the relationships among several aspects of TD and their relative relevance. The results of a network analysis confirmed that trait anhedonia relates to non-planning and cognitive impulsiveness, whereas trait dysthymia relates to motor impulsiveness, confirming that cognitive impulsiveness is an underlying characteristic of depression susceptibility, and trait dysthymia is a significant component connecting impulsiveness with trait sadness. Anhedonia and dysthymia seem to be distinct components in impulsive personality, and their management may aid the prevention of depression in this population. Moreover, Xu Y. et al. measured intolerance of uncertainty (IU) and electroencephalographic responses to uncertainty in trait-anxious (TA) and non-TA students to investigate whether mitigating anticipatory threat responses is a potential mechanism through which mindfulness may alleviate anxiety. In the predictable and unpredictable threat test, excessive anticipatory responses to unpredictable threats [IU, late positive potential (LPP), and reaction time (RT)] were high among TA students, along with low mindfulness. In uncertain threats, there were significant mediating effects of the LPP amplitude and RT on the relationship between mindfulness and anxiety. Shao et al. examined the factors influencing the effectiveness of online learning among 377 university students. A self-directed learning approach and attitude had a negative effect on the students' internet cognitive fatigue and a positive effect on their flow. Perceived learning ineffectiveness was positively influenced by internet cognitive fatigue and negatively influenced by the flow state. To enhance the effectiveness of online learning, online teachers may need to focus on improving students' self-directed learning awareness, attitude, and approach.

Living alone has been related to poor mental health, but large-scale epidemiological research on the association between living alone and depression and anxiety is scarce. Chen T. Y. et al. evaluated the correlation between living alone and psychiatric illness in a large population-based study. It revealed a statistically significant correlation between the two variables in married but not unmarried subjects. Given that living alone may be a risk factor for psychiatric illness in married individuals, it is important to enhance care delivery to married individuals living alone due to divorce, separation, or the death of their spouse in order to safeguard their physical and mental health. In a related study, Xu R. et al. used data from the Chinese Longitudinal Healthy Longevity Survey to examine the relationship between living arrangements and depressive symptoms among older adults (over 65). Living arrangements significantly correlated with the risk for depressive symptoms, with those living alone or in assisted living institutions expressing a higher risk than those living with household members. However, engaging in outdoor activities played a moderating role and reduced the risk of depression among older adults living in assisted living institutions.

## Author contributions

All authors listed have made a substantial, direct, and intellectual contribution to the work and approved it for publication.

